# In Their Own Words: Fears Expressed by People with Parkinson’s Disease in an Online Symptom Database

**DOI:** 10.3233/JPD-230305

**Published:** 2024-06-04

**Authors:** Sneha Mantri, Jennifer L. Purks, Daniel Kinel, Lakshmi Arbatti, Abhishek Hosamath, Allison Allen, Amy Amara, Karen Anderson, Lana M. Chahine, Shirley Eberly, Soania Mathur, David Standaert, David Oakes, Daniel Weintraub, Ira Shoulson, Connie Marras

**Affiliations:** a Department of Neurology, Duke University, Durham, NC, USA; b Department of Neurology, University of Rochester, Rochester, NY, USA; c Department of Neurology, Center for Health + Technology, University of Rochester Medical Center, Rochester, NY, USA; d Grey Matter Technologies, A Wholly Owned Subsidiary of Modality.ai, San Francisco, CA, USA; e Department of Neurology, University of Colorado Anschutz Medical Campus, Aurora, CO, USA; f Department of Psychiatry and Department of Neurology, Georgetown University, Washington, DC, USA; g Department of Neurology, University of Pittsburgh, Pittsburgh, PA, USA; h Department of Biostatistics and Computational Biology, University of Rochester, Rochester, NY, USA; i PD Avengers, Toronto, Canada; j Department of Neurology, University of Alabama at Birmingham, Birmingham, AL, USA; k Departments of Psychiatry and Neurology, Perelman School of Medicine, University of Pennsylvania, Philadelphia, PA, USA; l Edmond J. Safra Program in Parkinson’s Disease, University Health Network, University of Toronto, Toronto, Canada

**Keywords:** Parkinson’s disease, quality of life, qualitative

## Abstract

Parkinson’s disease (PD) carries substantial psychosocial burden. Using a database of responses by people with PD reporting up to five “most bothersome problems,” we identified 225 fear-based verbatims, which were organized using the framework method into 26 categories. Commonly-reported fears included uncertainty of progression (*n* = 60, 26.7%), fear of future cognitive impairment (*n* = 24, 10.7%) and fear of becoming a burden on others (*n* = 23, 10.2%). Fears in PD are wide-ranging and can constitute the most bothersome aspect of the condition. These data can be used to design interventions to lessen the psychosocial burden of PD.

## INTRODUCTION

Receiving a diagnosis of Parkinson’s disease (PD) has a profound impact on an individual’s mental health. In addition to well-described associations with anxiety and depression, a life-altering and incurable degenerative condition with uncertain prognosis may affect patients’ sense of self, alter their hopes for their future, and engender fear. These concerns have been previously described in other serious illnesses [1], but are infrequently elucidated in existing patient-reported outcome measures for people with PD (PwP). A better understanding of patient fears may help clinicians support PwP and their families through anticipatory guidance concerning specific symptoms, emotional support for existential concerns, and referral to mental health services if appropriate. It may also provide insight into the need to incorporate the impact of fears into assessment of quality of life and patient-reported outcome measures for PD research.

In this study, we assessed the range of fears reported by respondents to the Parkinson Disease Patient Report of Problems (PD-PROP), a series of questions asking PwP to submit up to 5 “most bothersome problems” and describe the impact on those problems on daily living. Participants are invited to complete the PD-PROP on a quarterly basis, forming a longitudinal database. The unique structure of the PD-PROP, with its longitudinal verbatim data capture, enables a deeper understanding of these critically important issues for people with PD [2]. By quantifying the domains and subthemes of fear as a “most bothersome problem,” this study provides preliminary data for the relative prevalence of different fears experienced by PwP.

## METHODS

### PD-PROP

The nature of PD-PROP has been previously described [2, 3]. Briefly, this is a series of questions incorporated into Fox Insight (FI, https://foxinsight.michaeljfox.org), an online, observational, longitudinal survey study sponsored by the Michael J. Fox Foundation for Parkinson’s Research (MJFF) [4]. The Fox Insight study and the PD-PROP module have received approval from the New England/WCG Institutional Review Board, and informed consent was obtained online from each participant. Participants are asked two questions: (1) What is the most bothersome problem for you due to your Parkinson’s disease? (2) In what way does this problem bother you by affecting your everyday functioning or ability to accomplish what needs to be done? Participant responses, termed “verbatims,” are entered online. Participants are asked to report up to five bothersome problems and are invited to respond to the PD-PROP on a quarterly basis.

### Data extraction, cleaning, and analysis

Data used in the preparation of this article were obtained from the FI database (https://foxinsight-info.michaeljfox.org/insight/explore/insight.jsp) in February 2020. For up-to-date information on the study, visit https://foxinsight-info.michaeljfox.org/insight/explore/insight.jsp. For the fear analysis, the PD-PROP database of over 25,000 responses was queried for verbatims matching the strings “fear of ___________” and “afraid of ___________,” as well as synonyms defined through a natural language processing (NLP) algorithm. The details of the NLP algorithm have been previously described [2] and are derived from the Unified Medical Language System ontologies [5] as well as NLP techniques such as word vectorization and sentence embedding [6, 7]. For this project, synonyms included “worry about,” “terrified of,” “anxious,” “nervous about”, “phobia”, and “dismay.” Two preliminary samples of 100 and 200 verbatims matching these words and strings were extracted. The analysis team (authors SM, JP, AA, DK, and CM) included three neurologists, one social worker, and one PwP. All members of the analysis team have professional and/or personal experience of PD, and the analysis team lead (author SM) has additional training in narrative medicine. Authors SM and CM reviewed the first 100 samples for clarity and requested the additional 200 samples to confirm that data saturation was achieved. For the purposes of this analysis, both samples were combined into a single primary group of 300. Of these, there were 9 word-for-word duplicates and one verbatim which referenced fears expressed by others (i.e., not the PwP). A further 65 verbatims did not have demographic information available. After removing these, the remaining 225 verbatims from 221 unique individuals were reviewed using the framework method for qualitative research [8]. This method is in contrast to other PD-PROP work which used a combination of human curation and machine learning to classify verbatims [2, 9]. For this study, each member of the analysis team independently reviewed the extracted verbatims and determined whether or not the verbatim expressed a fear, defined by the analysis team as “a distressing emotional state caused or triggered by an actual or perceived threat to self.” If so, they suggested categories, which were refined through iterative discussion to develop a codebook of domains and subthemes. Disagreement was resolved by consensus. Ultimately, the proportion of verbatims falling within each domain and subtheme was quantified.

## RESULTS

Demographic information about the participants is shown in Table 1. Most reported age of onset after 50 (*n* = 213, 96.4%) and had been living with PD for 5 years or less (*n* = 143, 64.7%). Twenty-six categories of fears were identified, arranged into seven domains (Table 2 and Supplementary Table 1): Relationships/Social, Dependence, Prognosis/Progression, Employment, Financial, Personal Safety, Specific Symptoms. The most commonly reported fear category was uncertainty around disease progression (*n* = 60/225, 26.7%). For instance, one participant wrote, “This is actually the worst problem with my PD = fear of progression and the future.” Uncertainty regarding day-to-day variability (e.g., “good days vs bad days”) or fear about death/dying were somewhat less common, reported by only 8 (3.5%) and 10 (4.4%) verbatims respectively. Twenty-three verbatims (10.2%) expressed fear of becoming a burden on family members, and 23 (10.2%) reported worries of becoming socially isolated as a result of their PD diagnosis. For instance, one participant expressed concern about the potential for “loss of spouse relationship. It dwells in a corner of my mind most of the time.” Another wrote “I want my life back ...  . Now I’m a prisoner having meds delivered to me.”

**Table 1:** Demographic Characteristics of Respondents (*N* = 221)

	*N* (%)
Sex:
Male	93 (42.1%)
Female	128 (57.9%)
Age of onset
<50	8 (3.6%)
50+	213 (96.4%)
Years since diagnosis:
<3	84 (38.0%)
3–5	59 (26.7%)
6–10	51 (23.1%)
11–14	17 (7.7%)
15+	10 (4.5%)
Race/ethnicity:
Black/African American	3 (1.4%)
Asian	3 (1.4%)
American Indian/Alaska Native	1 (0.5%)
Native Hawaiian/Pacific Islander	0
White/Caucasian	205 (92.8%)
Not Reported	9 (4.1%)

**Table 2:** Categories of Fears expressed in PD-PROP (*N* = 225). Quotes are copied verbatim from the participant’s response, in the format *Bothersome Problem [Impact on Function]*

Domain	Subthemes	Example Quotes	% (*n*)
Relationships/Social	Impact on family/being a burden	“My daughters worry about my health causing me to worry about them”“My fear of becoming a burden on my family. [We have a “high performing” family and I don’t want to be a drag on their potential]”	10.2% (23)
	Others’ perceptions	“tremor. [difficulty typing, using keyboard mouse, afraid what people think]”“the hardest part is that I’m still hiding this disease from everybody. But if I tell people I have Parkinson’s disease they’ll just stare at me and I don’t know if I’ll get the right support in at least in my workplace. It bothers me the most that this movement disorder makes people stare and not understand how difficult.”	10.2% (23)
	Social isolation	“Afraid to leave your home. [Always in isolation]”“Negative effect on relationships leading to isolation and depression.. [I haven’t changed, but people, understandably, treat me differently. I am perceived as a liability now, not an asset.]”	10.2% (23)
Dependence	Disability	“I will end up immobile and require professional assistance such as living in a care facility”“Fear of husbands health failing or of him dying and knowing that I can’t live alone, and knowing I am no longer independent.. [Causes depression, and then I do not function well, knowing that if I fall I cannot help myself up and neither can my husband. Fear makes me want to do nothing and it is a fight to keep going.]”	7.1% (16)
Prognosis/progression	Uncertainty about future	“This is actually the worst problem with my PD = fear of progression and the future”“PD consumes my thoughts. [I changed my outlook on life. Worry about future]”	26.7% (60)
	Fears of death/dying	“Thinking about dying when or if it will happen soon or how long do I really have left”“Possibility of short life span. [Scares me.]”	4.4% (10)
	Day-to-day variability	“It is difficult to know how far you can travel not knowing if it will be a good day”“I don’t have the stamina that I once had, and I don’t linked to age. I have to leave dinners and special sports occasions because I might crash.”	4.8% (11)
Employment	Work-related	“My work and office are very accommodating but I am not sure how long I can keep this up. I fear I am a liability so I wont let them file accidents reports when I fall.”“I lost my coaching job. [My medication at the time caused liability issues for my work.]”	4.4% (10)
Personal Safety	Safety	“Can’t drive fast out of fear of hitting with road divider or surrounding vehicles or persons, can’t take sharp turning, always feel afraid of loosing control in high speed”“R hand tremor. [Using kitchen knife is scary!]”	5.8% (13)
Specific symptoms	Cognitive impairment	“I fear the future and am terrified of getting dementia”“Fear of dementia and loss of spouse relationship. [It dwells in a corner of my mind most of the time, and makes me turn to God for comfort.]”	10.6% (24)
	Falling	“Frightful of falling while dressing and walking around clutter”“Apprehension when moving cautiously about –re: possibility of stumbling or tripping and falling. [When waling down stairs]”	10.6% (24)
	Cramps/spasms	“I’m always afraid of getting cramps when I stretch.”“Spasms, facial. [Afraid the spasms will occur in a meeting]”	4.8% (11)
	Unclassifiable motor symptoms	“Trepidation about doing anything involving movement”“physical discomfort, worry”	12.4% (28)
	Bladder symptoms	“Bladder control. [Nervous of leakage]”“incontinence. [afraid of messing self, pullups are expensive]”	4.8% (11)
	Constipation	“Constipation, lack of regular bowel movements. [Anxiety about being in wrong place when bowel movements might occur]”“Constipation ibs. [Constant worry about using bathroom]”	4.8% (11)
	Sleep-related	“Loss of sleep time, anxiety about being able to fall asleep”“I get sleepy middle of the day. [I’m afraid someone will catch me zoning out..]”	4.4% (10)

With regard to specific symptoms, fear of cognitive impairment (*n* = 24, 10.7%) and fear of falling (*n* = 16, 7.1%) were the most commonly reported. Other reported motor fears included fear of episodic symptom emergence; tremor (*n* = 5, 2.2%) and fear of cramps/spasms (*n* = 10, 4.4%). Non-motor fears included fear of poor nocturnal sleep quality or overwhelming daytime sleepiness (*n* = 8, 3.5%) and fear of GI distress (*n* = 9, 4%) such as constipation or nausea.

## DISCUSSION

In this online free-text database, we identified 26 categories of reported fears and worries as a “most bothersome problem” resulting from PD. Even though the participants reported a short duration of PD on average, many fears were expressed. This provides an important lesson for clinicians, who may focus on symptom management but fail to recognize that fear itself may be the most troublesome aspect of PD. Direct inquiry on this topic may open an important therapeutic avenue for patients.

The most commonly reported fears involved uncertainty around disease progression, fear of cognitive impairment, and concerns about becoming a burden on others. These findings are consistent with other data on quality of life and psychosocial burden in PD [10, 11] and in other chronic diseases [12, 13]. For instance, fear of progression was found to be prevalent in hospitalized people with PD [11] and appears to be associated with lower self-efficacy for managing chronic illness. Similarly, in a focus group study of people with primary brain tumors [14], themes included relational concerns, disease-specific symptoms, and existential concerns about the future. Interviews with people with dementia and their caregivers [15] identified discussions of the future and loss of independence as major themes. Acknowledging and addressing fear and uncertainty is a core tenet of neuro-palliative care [16, 17], and palliative interventions that allow patients to share their own stories with others [18] may reduce fears and increase hope, as noted by a few respondents in our study. Our findings on the range of fears experienced by people with PD can guide health care providers in inquiring about fears, connecting with patients and providing support to best care for patients with PD.

To our knowledge, this is the first large unstructured response study of fears in PD; the identified domains ranged from specific symptoms to existential concerns of the future. Major strengths of the PD-PROP database used in this study include the large nature of the PD-PROP and the open-ended question format, which allows participants to self-prioritize their “most bothersome problems.” This is in contrast to other qualitative studies of fears or anxieties, which traditionally rely on an face-to-face interactions to prompt participants and may inadvertently introduce a degree of bias into patient responses, wherein participants may respond in ways they feel socially appropriate [19]; by contrast, the online self-directed nature of PD-PROP enables participants to express themselves unmediated by the presence of others. In other words, the design of PD-PROP centers patient perspective, consistent with the growing recognition of the importance of patient engagement in research. Of note, the framework methodology used in this analysis differs from the machine-learning approach employed in other analyses of PD-PROP, demonstrating the value of triangulating qualitative and quantitative methods in understanding complex conditions like PD. Moreover, the fears identified in this sample can be validated against the wider PD-PROP dataset or other large online datasets such as internet forums or social media [20–22].

Nevertheless, some important caveats should be noted. Like other longitudinal cohorts of PwP, the FI cohort from which PD-PROP derives is predominantly English-speaking, non-Hispanic white, and well-educated [23]; targeted recruitment strategies have been implemented to increase enrollment of underrepresented populations [24], though these were not yet in place when the data was extracted for this analysis. Although data on fears in PD are limited, other cohorts of older adults reveal differing impact of race/ethnicity on fear of falling, with some data suggesting Black participants have a greater fear of falling [25] and other data suggesting that fear of falling is reduced in Black participants [26]. Additionally, the nature of the data extraction does not allow us to estimate the overall frequency of fears as most bothersome problems in PD, nor the correlation between fears and demographic or clinical characteristics. For instance, self-perception and existential anxieties may be impacted by age of onset [27], gender identity [28, 29], or disease duration [30]. Further, underlying mood disorder (e.g., depression or generalized anxiety) or medication use may also influence patient perceptions and fears. Additionally, fear types may change over time; the sample included in this project only included four instances where a single individual provided multiple fear-based verbatims. We did not perform a longitudinal analysis to identify how fear types and their priority as bothersome problems in PD may change over time. Future research, leveraging the larger FI study or other cohorts, may be able to better understand the longitudinal nature of fear in PD, as well as the interplay between patient self-report and these clinical/demographic characteristics. For instance, a natural language processing algorithm could extract fear-based topics more systematically from a large data source, providing a more comprehensive assessment of fear in this patient population.

In conclusion, fears in PD are wide-ranging and both existential and related to specific symptoms. In some patients, fear can itself be the most bothersome aspects of PD. Further analysis is needed to understand the demographic, clinical correlates and further impacts of PD fears. The range of fears quantified in the PD-PROP can be used to design educational materials and interventions to lessen the psychosocial burden of PD.

## Supplementary Material

Supplementary Material

## Data Availability

The data supporting the findings of this study are not publicly available due to privacy restrictions. The curated datasets derived from the verbatim responses are publicly available in FoxDEN at https://foxden.michaeljfox.org upon signing a data use agreement.
