# Supplementary material for: In Their Own Words: Fears Expressed by People with Parkinson’s Disease in an Online Symptom Database
Source: J Parkinsons Dis. 2024 Jun 4;14(4):865–72. doi: 10.3233/JPD-230305 (PMC11191490; doi:10.3233/JPD-230305)
Supplement: Supplementary Material [file jpd-14-jpd230305-s001.pdf]

Supplementary Material

In Their Own Words: Fears Expressed by People with Parkinson's Disease in an Online Symptom Database

Supplementary Table 1

Domain	Theme	Conceptual Boundaries	Example Quote	N=225 (included)	N=65 (excluded)	p for proportion test
**Relationships/ Social**	Impact on family/being a burden	Family members (spouse, children)	"My daughters worry about my health causing me to worry about them"	23	5	0.54
	Others' perceptions	Public perception, respondent embarrassment or distress	"tremor. [difficulty typing, using keyboard mouse, afraid what people think]"	23	5	0.54
	Social isolation	Isolated, staying away from others	"Afraid to leave your home. [Always in isolation]"	23	4	0.32
	Caregiving responsibilities to others	Person with PD caring for others<br>Exclude: care needs of person with PD	"Worry about my children (2 handicapped)"	3	1	0.9
**Dependence**	Disability	Disabled, professional caregiving	"I will end up immobile and require professional assistance such as living in a care facility"	16	0	0.03
	Quality of care	Poor quality of care from others	"being severely disabled with no one to care for me""	2	1	0.65
**Prognosis/ progression**	Uncertainty about future	Uncertainty, "the future," decline	"This is actually the worst problem with my PD = fear of progression and the future"	60	22	0.26
	Fears of death/dying	Death, dying	"Thinking about dying when or if it will happen soon or how long do I really have left"	10	0	0.08
	Day-to-day variability	Good days and bad days	"It is difficult to know how far you can travel not knowing if it will be a good day"	8	3	0.69
**Employment**	Work-related	Work, office, co-workers	"My work and office are very accommodating but I am not sure how long I can keep this up. I fear	9	1	0.34

			I am a liability so I wont let them file accidents reports when I fall.”			
**Financial**	Cost of care	Financial, money, payments/paying	“Expense! Travel to ongoing Physical Therapy, Dr visits, paying for parking, food out of town, hospital stays, supplements, adaptive clothing, shoes, uStep walker and on and on!”	5	0	0.23
**Personal Safety**	Safety	Personal injury from falls, accidents, etc.	“can't drive fast out of fear of hitting with road divider or surrounding vehicles or persons, can't take sharp turning, always feel afraid of loosing control in high speed”	12	1	0.19
Specific Symptoms: Motor	Falling	Falls, fear of falling	“Frightful of falling while dressing and walking around clutter”	16	9	0.06
	Cramps/spasms	Tightness, cramp, spasm	“I'm always afraid of getting cramps when I stretch.”	10	1	0.28
	Tremor	Shaking, spilling, tremor	“eating in public. [worry about spilling food]”	5	3	0.3
	Choking	Swallowing difficulty, choking	“Swallowing, fear of choking”	4	3	0.1
	Unclassifiable motor symptoms	Any other motor symptoms	“Trepidation about doing anything involving movement”	21	7	0.73
Specific symptoms: Non-Motor	Cognitive impairment	Dementia or cognitive impairment in the future Exclude: current cognitive impairment or dementia	“I fear the future and am terrified of getting dementia”	24	10	0.35
	Constipation	GI, constipation, bowel	“Constipation, lack of regular bowel movements. [Anxiety about being in wrong place when bowel movements might occur]”	9	2	0.73
	Sleep-related	Sleep, dream enactment	“loss of sleep time, anxiety about being able to fall asleep”	8	2	0.85
	Bladder symptoms	Bladder, incontinence, leakage	“Bladder control . [Nervous of leakage]”	7	4	0.26
	Dizziness	Dizziness, lightheadedness, blood pressure	“low blood pressure due to medication. [afraid to fly in	4	1	0.9

			airplane (lost conscienceness [sic] one time)]”			
	Pain/paresthesia	Pain, tingling	“Weird feelings in body; tingling/numbness/electric shocks over body/inner tremor.. [I am distressed by these feelings. I do not know if they are PD, signs of a stroke or just anxiety.”	3	0	0.35
	Drooling	Salivation, drooling	“excessive salivation. [potential embarrassment]”	3	0	0.28
	Hyposmia	Loss of smell	“Loss of smell. [It’s a concern to not be able to smell something burning or unfamiliar in case there were a fire or other potential danger]”	2	1	0.65
	Psychosis	Hallucination, delusion	“I worry about what my life will be like as the disease progresses, especially regarding hallucinations and dementia. . [It makes it difficult to do long term planning and I find that I frequently second guess myself when I get confused or forget something or think I see a bug.]”	2	1	0.65

Bonferroni correction for multiple comparisons: significance threshold at $p<0.002$
